# Identification of Altered Metabolic Pathways in Plasma and CSF in Mild Cognitive Impairment and Alzheimer’s Disease Using Metabolomics

**DOI:** 10.1371/journal.pone.0063644

**Published:** 2013-05-20

**Authors:** Eugenia Trushina, Tumpa Dutta, Xuan-Mai T. Persson, Michelle M. Mielke, Ronald C. Petersen

**Affiliations:** 1 Department of Neurology, Mayo Clinic, Rochester, Minnesota, United States of America; 2 Department of Pharmacology and Experimental Therapeutics, Mayo Clinic, Rochester, Minnesota, United States of America; 3 Center for Translational Science Activities Metabolomics Core Facility, Mayo Clinic, Rochester, Minnesota, United States of America; 4 Division of Endocrinology and Endocrine Research Unit Rochester, Mayo Clinic, Rochester, Minnesota, United States of America; 5 Department of Health Sciences Research, Division of Epidemiology, Mayo Clinic, Rochester, Minnesota, United States of America; Rush University, United States of America

## Abstract

Alzheimer’s Disease (AD) currently affects more than 5 million Americans, with numbers expected to grow dramatically as the population ages. The pathophysiological changes in AD patients begin decades before the onset of dementia, highlighting the urgent need for the development of early diagnostic methods. Compelling data demonstrate that increased levels of amyloid-beta compromise multiple cellular pathways; thus, the investigation of changes in various cellular networks is essential to advance our understanding of early disease mechanisms and to identify novel therapeutic targets. We applied a liquid chromatography/mass spectrometry-based non-targeted metabolomics approach to determine global metabolic changes in plasma and cerebrospinal fluid (CSF) from the same individuals with different AD severity. Metabolic profiling detected a total of significantly altered 342 plasma and 351 CSF metabolites, of which 22% were identified. Based on the changes of >150 metabolites, we found 23 altered canonical pathways in plasma and 20 in CSF in mild cognitive impairment (MCI) vs. cognitively normal (CN) individuals with a false discovery rate <0.05. The number of affected pathways increased with disease severity in both fluids. Lysine metabolism in plasma and the Krebs cycle in CSF were significantly affected in MCI vs. CN. Cholesterol and sphingolipids transport was altered in both CSF and plasma of AD vs. CN. Other 30 canonical pathways significantly disturbed in MCI and AD patients included energy metabolism, Krebs cycle, mitochondrial function, neurotransmitter and amino acid metabolism, and lipid biosynthesis. Pathways in plasma that discriminated between all groups included polyamine, lysine, tryptophan metabolism, and aminoacyl-tRNA biosynthesis; and in CSF involved cortisone and prostaglandin 2 biosynthesis and metabolism. Our data suggest metabolomics could advance our understanding of the early disease mechanisms shared in progression from CN to MCI and to AD.

## Introduction

Alzheimer’s Disease (AD) is a progressive neurodegenerative disorder that involves loss of memory and cognitive abilities. The molecular mechanisms of AD remain elusive. Neuritic plaques that contain β-amyloid (Aβ) aggregated peptides and intracellular neurofibrillar tangles consisting of hyperphosphorylated microtubule-associated *tau* protein are two major hallmarks of AD [1], [2]. Accumulating research evidence suggests that pathophysiological changes associated with AD begin at least 10 to 25 years before dementia onset [3]–[7]. Indeed, the FDA-approved drugs for AD do not provide cure, most likely because they are administered too late in the disease process. Clinical trials conducted over 18 months utilizing Aβ-clearing monoclonal antibodies failed to slow cognitive and functional decline in AD patients despite the maintenance of amyloid burden and actual decrease in phospho-tau (p-tau) levels in cerebrospinal fluid (CSF) [8]–[10]. Thus, there is an urgent need to identify alternative disease mechanisms and associated biomarkers that can help to diagnose AD in the preclinical and early clinical (i.e., mild cognitive impairment (MCI)) stages [11].

Research conducted in multiple cellular and animal models strongly suggests that AD pathophysiology involves early changes in functionally connected networks across many brain regions that are not limited to cognition and learning [12]–[19] However, currently available biomarkers are limited to the measurements of tau, p-tau, and Aβ levels in CSF and plasma [20]–[22] that represent rather narrow hypothesis-driven biomarker development. Combining data on tau and Aβ levels with the results obtained using advanced brain imaging techniques comprising of computed tomography, nuclear magnetic resonance imaging, and single photon or positron emission computed tomography could enhance AD diagnosis [23]. Nevertheless, there is clearly a need for broader biomarker investigations that should lead to better understanding of early disease mechanisms. Therefore, the identification of markers in readily available biofluids suitable for large-scale clinical applications using methods that could provide information on global changes in the cellular networks with high accuracy and reduced cost is of great importance.

Metabolomics is a powerful tool that studies perturbations in the metabolome, which reflects genomic, transcriptomic and proteomic changes and represents an accurate biochemical phenotype of the organism in health and disease [24]. Metabolomic profiling can be done relatively easily in peripheral tissues, CSF or plasma, making this approach valuable for clinical application. Based on the type of utilized analytical platform, non-targeted metabolomics facilitates establishing of broad metabolic profiles in samples including identification of novel metabolites that can be used as biomarkers. This approach detects changes in the levels of hundreds of metabolites providing valuable information on the alterations in multiple metabolic networks. Targeted metabolomics offers data on quantitative changes in specific classes of metabolites of interest such as amino acids, lipids, fatty acids and others. Using a targeted metabolomics approach, we previously identified particular pathways and a mutation-specific panel of biomarkers in the brain tissue of three transgenic mouse models representing familial AD (FAD) [25] that were affected early in disease process, prior to the formation of amyloid plaques and the onset of memory impairment. We demonstrated that changes in brain energetic and mitochondrial function were among the underlying mechanisms in all three FAD animal models [25]. Recent investigations conducted in human samples have identified changes in a number of metabolites in CSF or plasma that also correlated with AD severity validating metabolomics as a useful tool to study the disease progression [17], [18], [26]–[31]. However, none of these studies applied a non-targeted metabolomics approach to identify global changes in metabolites and canonical metabolic pathways perturbed across the AD clinical spectrum (e.g., cognitively normal, MCI, and AD) in both CSF and plasma from the same individuals. Since plasma represents a non-invasive, inexpensive, and acceptable source for repeated measures, the demonstration that metabolic profiles in plasma resemble the profiles in CSF, which most closely reflects brain-specific changes, is of great importance. Here, we present the results of the study conducted in the cohort of MCI, AD and cognitively normal (CN) subjects enrolled in the longitudinal Mayo Clinic Study of Aging (MCSA) and Mayo Clinic Alzheimer Disease Research Center (ADRC) utilizing a non-targeted ultra-performance liquid chromatography coupled to time-of-flight mass spectrometry (UPLC-ToF-MS)**–** based metabolomics.

## Materials and Methods

### Subjects and Sample Collection

The study was approved by the Institutional Review Boards of the Mayo Clinic and Olmsted Medical Center. Written informed consent was obtained for all participants. Forty-five subjects were enrolled in the study. Of these, 14 CN subjects, 13 amnestic MCI, and 11 AD dementia patients were enrolled and followed in the MCSA, a population-based epidemiologic study of normal ageing and MCI in individuals aged 70–90 years in Olmsted County, Minnesota [32] (Table 1). The remaining 2 amnestic MCI subjects, 4 AD patients, and one CN individual were enrolled in the ADRC. ADRC recruitment is drawn from individuals seeking medical care at the Mayo Clinic. Both the MCSA and ADRC are longitudinal studies that include serial clinical and cognitive assessments. The clinical diagnosis at each visit was made at weekly consensus conferences that include neurologists, neuropsychologists, a neuropsychiatrist, and study coordinators. Control subjects were asymptomatic CN volunteers. Demographic characteristics and clinical diagnosis of studied subjects are summarized in Table 1. Criteria for the diagnosis of amnestic MCI were outlined in [33] and included: (i) memory complaint documented by the patient and collateral source; (ii) impairment in 1 or more of the 4 cognitive domains (memory, executive functioning/attention, visuospatial, or language); (iii) essentially normal functional activities of daily living; and (iv) absence of dementia. In general, the amnestic MCI determination is made when the memory measures fall 1.0–1.5 SD below the means for age and education appropriate individuals in our community; however, rigid cutoffs on psychometric scores were not used to establish the diagnosis of amnestic MCI which was made on clinical grounds. The diagnosis of dementia was made using DSM-IV criteria [34], and the diagnosis of AD was made using established criteria [35]. Subjects were considered to be CN if they performed within the normative range and did not meet criteria for MCI or dementia. CSF was collected from individuals in the fasting state by a lumbar puncture while lying on their side or in a seated position. Plasma was collected in EDTA tubes from individuals after an overnight fast using standard venipuncture procedures. Both CSF and plasma were immediately centrifuged and aliquoted according to standard procedure established at the clinical laboratories of Mayo Clinic. CSF and plasma were stored in aliquots at −80°C until the date of analysis.

**Table 1:** Characteristics of Study Participants.

Characteristics	MCI	AD	CN
n	15	15	15
**Age (years)** 1	80.4±4.2	82.7±4.2	78.6±3.5
**Race** 2	White (n = 15)	White (n = 14)	White (n = 14)
		Asian (n = 1)	Asian (n = 1)
**Male, %**	73	80	67
**Education (years)** 1	14.1±3.3	13.9±3.2	14.8±3.0
**Family History of AD**	0 positive	4 positive	3 positive
	2 unknown	3 unknown	
**Family History of Dementia** 3	2 positive	8 positive	7 positive

1 Data are mean ± SD for all participants.

2 All participants except of one (unknown ethnicity) were non-Hispanic or Latino.

3 Family history of dementia including AD.

### Sample Preparation for Metabolomic Profiling

The metabolite extraction method was performed as described previously [36] with minor modifications in the method. Plasma and CSF samples (100 µL) were thawed on ice at 4°C followed by deproteinization with methanol (1∶4 ratio of plasma to methanol) and vortexed for 10 s, followed by incubation at −20°C for 2 h. Prior to deproteinization, 4 µL of an internal standard solution of ^13^C_6_-Phenylalanine (247 ng/µL) was added to each plasma, CSF, and quality control (QC) samples to monitor the recovery of extracted metabolites. The samples were centrifuged at 18000 g for 20 min at 4°C. The supernatants were lyophilized (Savant, Holbrook, NY) and stored at −20°C prior to analysis. The samples were reconstituted in running buffer and analyzed within 24 hrs. Metabolite separation in plasma and CSF was achieved using an Acquity UPLC system (Waters, Milford, MA) with both hydrophilic interaction chromatography (HILIC) (ethylene-bridged hybrid 2.1×150 mm, 1.7 mm; Waters) and reversed-phase liquid chromatography C18 (RPLC) (high-strength silica 2.1×150 mm, 1.8 µm; Waters). For each column, the run time was 20 min at a flow rate of 400 µL/min. Reverse-phase chromatography was performed using 99% solvent A (5 mmol/L NH4 acetate, 0.1% formic acid, and 1% acetonitrile) to 100% solvent B (95% acetonitrile with 0.1% formic acid). The gradient was 0 min, 0% B; 1 min, 0% B; 3 min, 5% B; 13.0 min, 100% B; 16 min, 100% B; 16.5 min, 0% B; and 20 min, 0% B. The hydrophilic interaction chromatography gradient was as follows: 0 min, 100% B; 1 min, 100% B; 5 min, 90% B; 13.0 min, 0% B; 16 min, 0% B; 16.5 min, 100% B; and 20 min, 100% B. The injection volume of each sample was 5 µL and column was maintained at 50°C. Each sample was injected and analyzed in duplicate. QCs and standards were run at the beginning and the end of each sequence to monitor shift in the retention time on the column.

### Mass Spectrometry

A 6220 ToF-MS (Agilent Technologies) was operated in both positive and negative electrospray ionization (ESI) modes using a scan range of 50**–**1,200 m/z. The mass accuracy and mass resolution were 5 parts per million (ppm) and 20,000 ppm, respectively. The instrument settings were as follows: nebulizer gas temperature 325°C, capillary voltage 3.5 kV, capillary temperature 300°C, fragmentor voltage 150 V, skimmer voltage 58 V, octapole voltage 250 V, cycle time 0.5 s, and run time 15.0 min.

### Data Preprocessing

All ToF-MS raw data files were converted to compound exchange File (CEF) format using Masshunter DA reprocessor software (Agilent Technologies Inc). Chromatography and centroided MS data were aligned to generate a single data matrix consisting of retention time (RT), mass-to-charge (m/z), and normalized ion intensity for each detected peak in individual samples. Mass Profiler Professional (Agilent Technologies Inc) was used for data alignment and to convert each metabolite feature (m/z×intensity×time) into a matrix of detected peaks versus compound identification [36]. Each sample was normalized to the median of the baseline and log 2 transformed. Default settings were used with the exception of signal-to-noise ratio threshold (3), mass limit (0.0025 units), and time limit (9 s) [36]. The resulting metabolites were identified against the METLIN metabolite database using a detection window of ≤5 ppm. Putative identification of each metabolite was made based on mass accuracy (m/z) Chemical Abstracts Service (CAS), Kyoto Encyclopedia of Genes and Genomes (KEGG), Human Metabolome Project (HMP) database, and LIPID MAPS identifiers [37], [38]. Method performance was evaluated for the ten metabolite standards with respect to limit of detection, linearity, reproducibility, and mass accuracy (<5 ppm); coefficient of variation was ≤5% [36].

### Statistical Analysis

**Figure 1:**
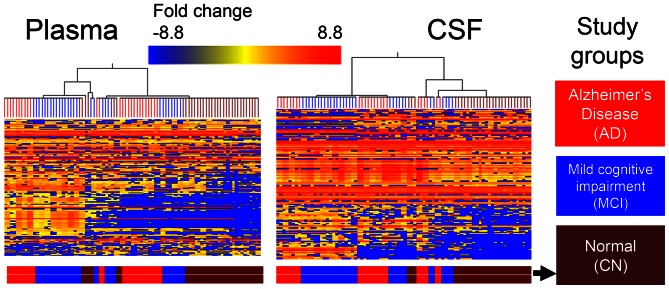
Heat map analysis of metabolites in plasma and CSF samples from CN, MCI and AD patients. Metabolite perturbations were calculated based on the median for each metabolite level of three independent biological replicates of plasma and CSF samples from each study participant. Each row represents a metabolite, and each column depicts a subject. The study groups are color-coded as follows: AD is denoted in red, MCI is denoted in blue, and CN is denoted in maroon. The fold change in metabolite levels is color-coded: red pixels, up regulation; blue, down regulation; yellow, no significant change.

**Figure 2:**
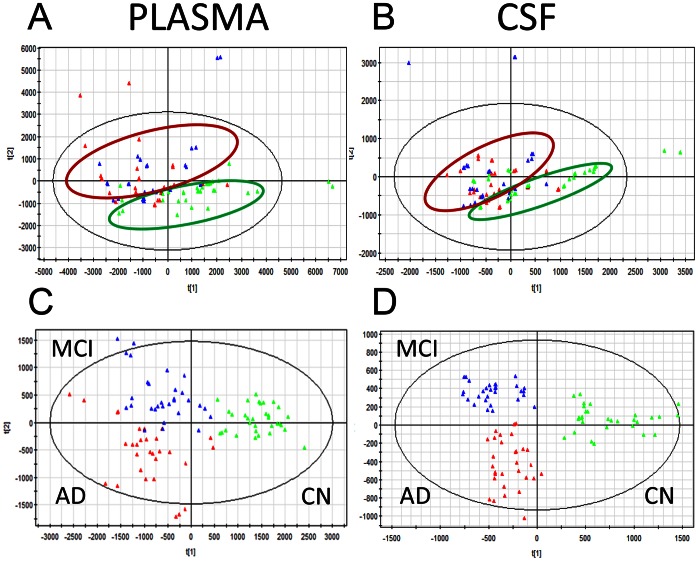
Plasma and CSF samples have distinct metabolomic profiles between AD, MCI and CN groups. Two-dimensional score plots of unsupervised principal component analysis (PCA) of the plasma (**A**) and CSF (**B**) samples, and orthogonal two partial least squares-discriminant analysis (O2PLS-DA) of plasma (**C**) and CSF (**D**) samples from AD (red), MCI (blue) and CN (green) patients. Each sample is labeled with a triangle.

**Figure 3:**
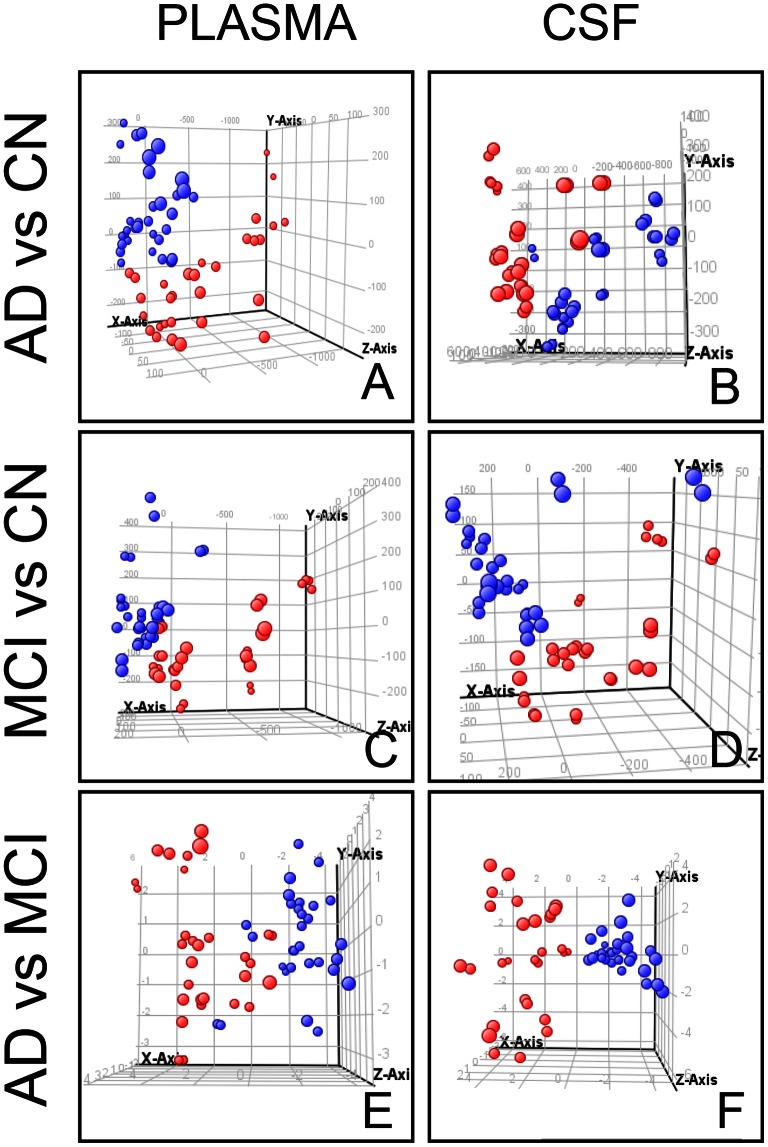
Unsupervised Principal Component Analysis (PCA) of plasma and CSF samples from CN, AD and MCI subjects. Each dot corresponds to an individual sample. (**A, B**): AD – red; CN – blue; (**C, D**): MCI – red; CN – blue; (**E, F**): AD – red; MCI- blue.

The metabolites detected in at least ≥50% of the samples in any of three study groups (AD, MCI, and CN) were selected for differential expression analyses. Univariate statistical analysis, one-way analysis of variance (ANOVA), was used to find the differentially expressed metabolites across the three study groups. Hierarchical cluster analysis of metabolites was performed to reveal associations between replicate biological samples within a group based on the similarity of their mass abundance profiles. Hierarchical cluster analysis was performed on the log-2**–**transformed, one-way ANOVA data set. A heat map was generated wherein each column depicts a sample and each row represents a metabolite, with the relative change color coded (Fig. 1). Unpaired t-test analysis was performed for comparison of two groups (e.g., AD vs. CN; MCI vs. CN, and AD vs. MCI) in both plasma and CSF. To address false discovery rates (FDR) from multiple comparisons, Benjamini–Hocherberg correction (0.05) was applied on each pair of analysis. The mean-centered, paretoscaled and log2 transformed data were then introduced into the SIMCA-P 11.5 software (Umetrics, Umeå, Sweden) for multivariate statistical analysis. Principal components analysis (PCA) using Pareto-scaled data was performed to reduce the dimensionality of the data and to reveal any clustering of the three study groups (AD, MCI and CN) in an unsupervised manner (Fig. 2). Three PCA analyses were conducted comparing two groups at a time (AD vs. CN; MCI vs. CN; AD vs. MCI) for plasma and CSF separately (Fig. 3). Orthogonal projections to latent structures discriminant analysis (O2PLS-DA), a supervised pattern recognition approach, was utilized to construct a predictive model to compare and evaluate between plasma or CSF depicting disease condition and/or progression based on the differential metabolites accountable for AD. To avoid the over fitting of the models, the OPLS-DA model was validated by an iterative 7-round cross-validation with one seventh of the samples being excluded from the model and blind prediction test in which the data set was randomly divided into training set (70%) and test set (30%). The model built on the training set was applied to build the classification model to predict the class membership of the test set.

### Pathway Analysis

The differentially expressed metabolites (p<0.05 with FDR of 0.05) were analyzed for pathway enrichment using MetaCore (Genego, St. Joseph, MI) [39]. Metabolite identifiers (CAS and KEGG) were used for each metabolite including name and molecular weight in addition to fold change and differential P value. The P value from the hypergeometric test, generated by Metacore, represents the enrichment of certain metabolites in a pathway. A P value ≤0.05 is indicative of significant enrichment. The ratio of significantly changed metabolites in the pathway to total number of metabolites in a pathway was also calculated. A FDR of 0.15 was also applied for pathway enrichment [36].

## Results

### Metabolomics Profiling in Plasma and CSF by UPLC-ToF-MS in AD, MCI and CN Groups

For analysis of the individual metabolite fingerprints, we employed non-targeted UPLC-ToF-MS **–**based comprehensive metabolomic profiling to determine changes in metabolites associated with AD severity in the CSF and plasma samples from the same patients diagnosed with amnestic MCI or AD and CN individuals. For each sample, reversed-phased and hydrophilic interaction chromatography (HILIC) UPLC-ToF-MS analyses were applied both in the positive and negative electron spray ionization (ESI) modes to increase the number of detected metabolite ions. Metabolic profiling detected a total of 342 and 351 (*P*≤0.05) metabolites in the plasma and CSF, respectively, of which 22% were identified (Tables S1 and S2). There were 65 plasma and 74 CSF metabolites (*P*≤0.05) detected and identified in three study groups (Table S1 and S2, top metabolites in bold font). Metabolites detected in plasma and CSF were confirmed based on a comparison with known standards and retention time listed in Table S3. The identification of the other metabolites was based on accurate mass in database searches with the METLIN metabolite database using a detection window of ≤5 ppm. A heat map, generated based on the identified and unidentified metabolites for plasma and CSF (Fig. 1), revealed considerable differences between CN, MCI and AD groups. The metabolites shown in red color are up regulated and those in blue are down regulated (Fig. 1).

The heat map of plasma and CSF demonstrated that CN group is predominantly clustered separately from AD and MCI due to their inherent differences in metabolic changes. Some overlap in MCI and AD individuals is anticipated based on the disease progression and is reflected in Fig. 1. Plasma metabolites with significantly elevated levels in AD individuals in comparison to MCI and CN included assymmetric dimethylarginine, glycerophosphoethanolamine, 2β,3β-dihydroxy-6-oxo-5α-chol-7-en-24-oic acid, 5-octadecylenic acid, hippuric acid, 5,7-nonadienoic acid, vitamin-D derivatives, propionylglycine methyl ester, glucopyranoside, 1-methyladenosine glycoursodeoxycholic acid. In contrast, levels of indoleacrylic acid, succinic anhydride, tryptophan, 3E,13Z-octadecadien-1-ol, 2-oxo-4-hydroxy-hexanoic acid, biliverdin IX, phenylalanine, bilirubin, 2-methylbutyrylglycine, and aminocyclohexanecarboxylic acid were significantly reduced (Table S1). Metabolomic analysis in CSF samples revealed similar changes in many of the metabolites along with identification of new compounds that were not detected in plasma such as pyruvic acid, glutamic acid dibutyl ester, N-acetyl-α-neuraminic acid, methyl-L-lysine, and bile acid derivatives (Table S2). Levels of multiple amino acids and metabolites of TCA (Krebs) cycle in addition to methylglyoxal, pyrimethamine, pyroglutamic acid, L-aspartic acid b-semialdehyde, fumaric acid, 3-methyl-2-oxobutanoate, hydroxy-L-threonine, and oxaloglutarate were specifically decreased in CSF suggesting altered metabolism in comparison to CN individuals (Table S2).

### Multivariate Analysis of AD, MCI and CN Study Groups

To determine whether the metabolite fingerprints in fasting plasma and CSF differed between CN, MCI and AD subjects in our metabolomics approach, we first evaluated separation between experimental groups using unsupervised principal component analysis (PCA) (Fig. 2 A, B). Strong group separation was achieved in both plasma and CSF between all three groups (Fig. 2 A, B), and in two-group comparison: AD vs. CN; MCI vs. CN, and MCI vs. AD (Fig. 3). Further analysis using orthogonal two component PLS-DA (O2PLS-DA) models demonstrated robust group separation between all three groups (AD, MCI and CN) for both plasma (Fig. 2C) and CSF (Fig. 2D). To ensure that the calculated models were reliable and the observed clustering was not due to chance, we performed an internal validation using 7-fold cross-validation [40]. The calculated goodness of fit (R2Y) was 0.639 for plasma, and 0.791 for CSF, and the goodness of prediction (Q2Y) 0.499 for plasma and 0.717 for CSF, respectively, which emphasizes the robustness of the model. Despite clear group separation for both fluids, separation in CSF was more robust (Fig. 2A–D).

### Altered Metabolites and Canonical Pathways in CSF and Plasma of MCI Subjects vs. CN

We identified 109 (*P*≤0.05) metabolites in plasma (Table S4) and 111 (*P*≤0.05) metabolites in CSF (Table S5) that were significantly affected in MCI patients vs. CN. Among metabolites that had 2–5 fold increase in plasma of MCI group were multiple derivatives of vitamin D3, phophatidylethanolamine, N-acetylcadaverine, hippuric acid, a-hydroxyisovalerate, and hydroxyhydroquinone (Table S4). Metabolites that were 2–3 fold reduced included 6-hydroxy-2-hexynoic acid, 8R-hydroxy-9Z-octadecenoic acid, 1-aminocyclohexanecarboxylic acid, guaifenesin, 5,7-nonadienoic acid, biliverdin IX, 2-methylbutyrylglycine, bilirubin, propionylglycine methyl ester, multiple derivatives of vitamin D3, and glycerophospholipids (Table S4).

Metabolites with 2–6 fold increase in CSF of MCI patients vs. CN group included succinic anhydride, citraconic acid, 2-furoic acid, threo-Isocitric acid, pyruvic acid, methionine, ethosuximide, and p-aminobenzoic acid (Table S5). CSF metabolites reduced 2–16 fold in MCI vs. CN included: acetoacetic acid, fumaric acid, gualenate, lorazepam, a-ketoglutarate, 2-methylbutyrylglycine, multiple derivatives of vitamin D3, tranexamic acid, and diallyl sulfide (Table S5). Notably, 2-methylbutyrylglycine and different analogs of vitamin D3 were decreased to a similar extent in both the plasma and CSF (Tables S4 and S5).

**Figure 4:**
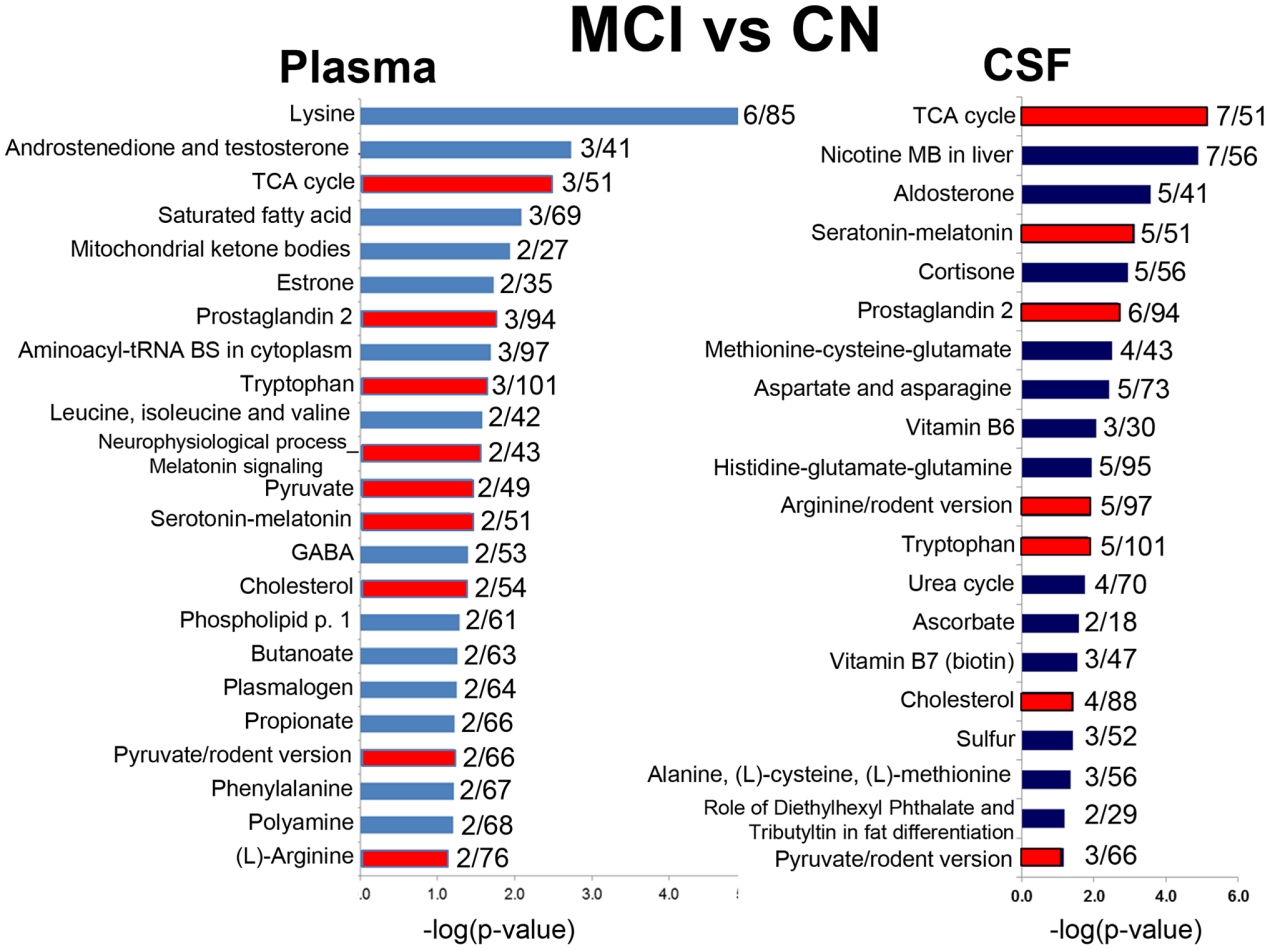
Altered metabolic pathways and process networks in plasma and CSF of MCI vs. CN subjects. The significance of the pathways was evaluated using *P* values and false discovery rate <0.05. Pathways that are affected in both fluids are colored in red. The ratio depicts the number of affected metabolites to the total number of metabolites in the pathway. TCA – tricarboxylic acid (Krebs) cycle; GABA - γ-Aminobutyric acid; MB – metabolism.

Additional analysis of the identified altered metabolites showed that 23 canonical pathways (*P*≤0.05) in plasma and 20 (*P*≤0.05) in CSF were perturbed in MCI patients (Fig. 4) with FDR <0.05. About 30% of the pathways significantly altered in CSF were also altered in plasma (Fig. 4, red). Common pathways that were affected in both fluids were related to the amino acid metabolism, neurotransmitter metabolism, mitochondrial function and Krebs cycle, fatty acid biosynthesis, and lipid biosynthesis and metabolism. Lysine metabolism (P≤0.00001) in plasma and TCA cycle (P≤0.00001) in CSF were affected to the greatest extent.

### Altered Metabolites and Canonical Pathways in CSF and Plasma from AD Subjects vs. CN

We have identified 154 (*P*≤0.05) metabolites in plasma (Table S6) and 150 (*P*≤0.05) in CSF (Table S7) with significant changes in AD vs. CN subjects. In plasma, the following metabolites were increased 2–5 fold: 8-amino-7-oxononanoate, phosphatidylethanolamine, ethosuximide, 2-methylbutyrylglycine, 1,3-dipropyl-8-cyclopentylxanthine, dihydrofissinolide, methionine, histidine, lysine, L-urobilin, p-hydroxyaniline, and 2-oxo-4-hydroxy-hexanoic acid (Table S8). Levels of the following metabolites were 2–3 fold decreased: 6-α-hydroxycastasterone, a-hydroxyisobutyrate, norcodeine, valeryl salycilate, biliverdin IX, hypoxanthine, and vitamin D3 derivatives (Table S6). In CSF, 2–3 fold increase was observed in the levels of succinic anhydride, arecoline, N-methyl-L-lysine, dehydroascorbic acid, citraconic acid, and pyruvic acid; while levels of 7-hydroxy tetranor iloprost (Ventavis), prostaglandin E2-α dimethyl amine, 2-octenedioic acid, acetoacetic acid, methyl 7-deshydroxypyrogallin-4-carboxylate, 2-hydroxy-3-(4-methoxyethylphenoxy)-propanoic acid, anthracene, pyrogallin, 1-hydroxy-2-naphthoic acid, vitamin D3 derivatives, and methoxsalen metabolites were 2–3 fold decreased (Table S7). Levels of amino acids were similarly increased in both plasma and CSF, while levels of vitamin D3 derivatives were equally reduced in both fluids (Tables S6, S7).

**Figure 5 pone-0063644-g005:**
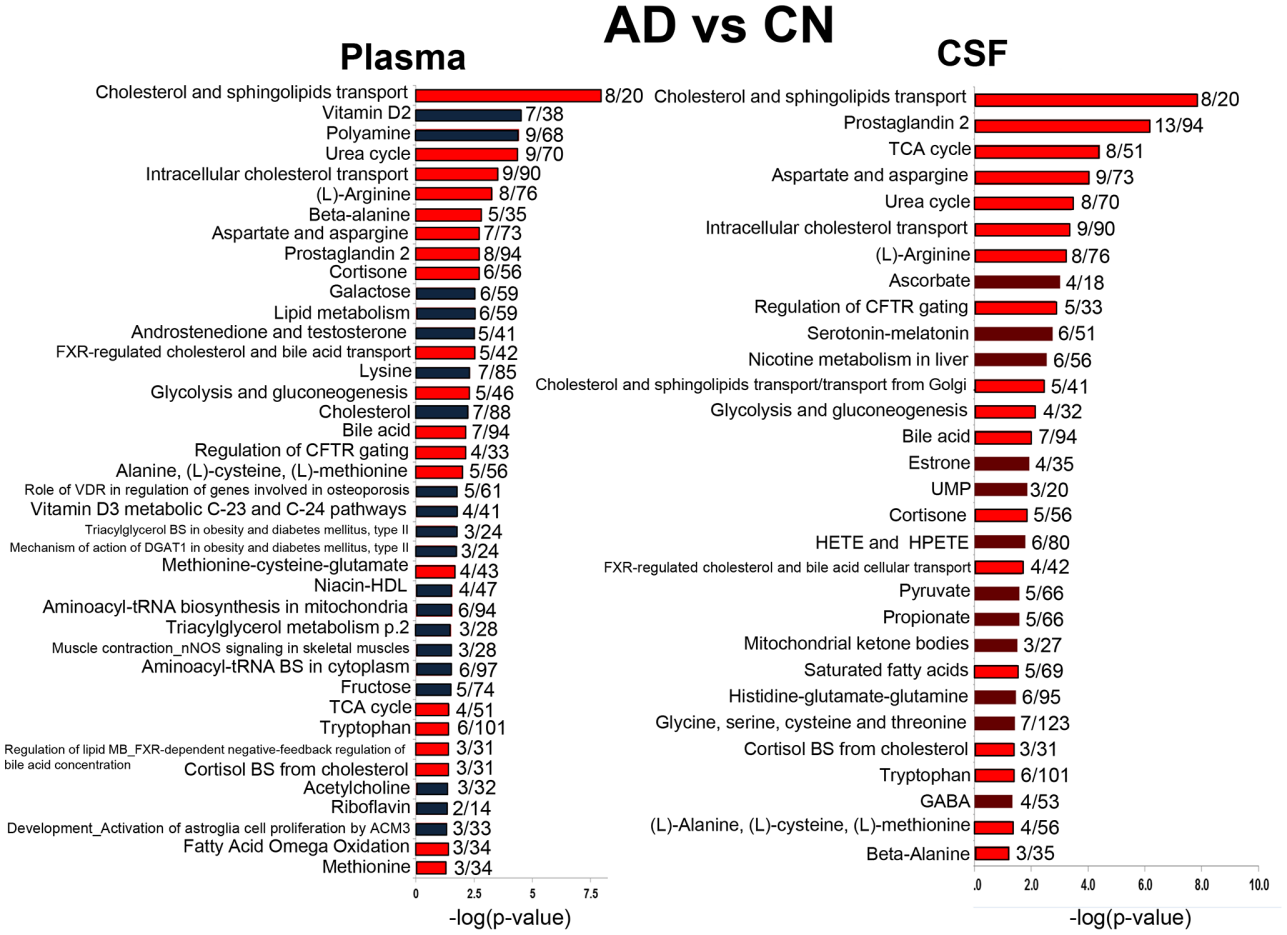
Canonical pathways and process networks affected in plasma and CSF of AD vs. CN subjects. The significance of the pathways was evaluated using *P* values and false discovery rate <0.05. Pathways that are affected in both fluids are colored in red. The ratio depicts the number of affected metabolites to the total number of metabolites in each pathway. FXR - farnesoid X receptor; CFTR - cystic fibrosis transmembrane conductance regulator; VDR - vitamin D receptor; DGAT1 - diacylglycerol acyltransferase 1; nNOS – neuronal nitric oxide synthase; UMP - Uridine monophosphate; HETE - hydroxyeicosatetraenoic acid; HPETE - hydroperoxyeicosatetraenoic acid; BS – biosynthesis.

Canonical pathways altered in plasma and CSF of AD vs. CN subjects are presented in Fig. 5. There were 40 pathways (*P*≤0.05) in plasma and 30 (*P*≤0.05) in CSF that were significantly affected; 60% of the pathways were perturbed in both CSF and plasma (Fig. 5, red). Pathways affected to the highest extent in plasma included cholesterol and sphingolipids transport, vitamin D2 (ergocalciferol) metabolism, polyamine metabolism, and urea cycle. Cholesterol and sphingolipids transport, prostaglandin 2 (PGE2) biosynthesis and metabolism, TCA cycle, and aspartate and asparagine metabolism were among the most affected in CSF of AD subjects (Fig. 5). Pathways that were equally affected in CSF and plasma were related to lipid and cortisone biosynthesis and metabolism, mitochondrial function and energy production, urea cycle, bile acid metabolism, and amino acid biosynthesis and metabolism.

### Altered Metabolites and Canonical Pathways in CSF and Plasma of AD Subjects vs. MCI

**Figure 6:**
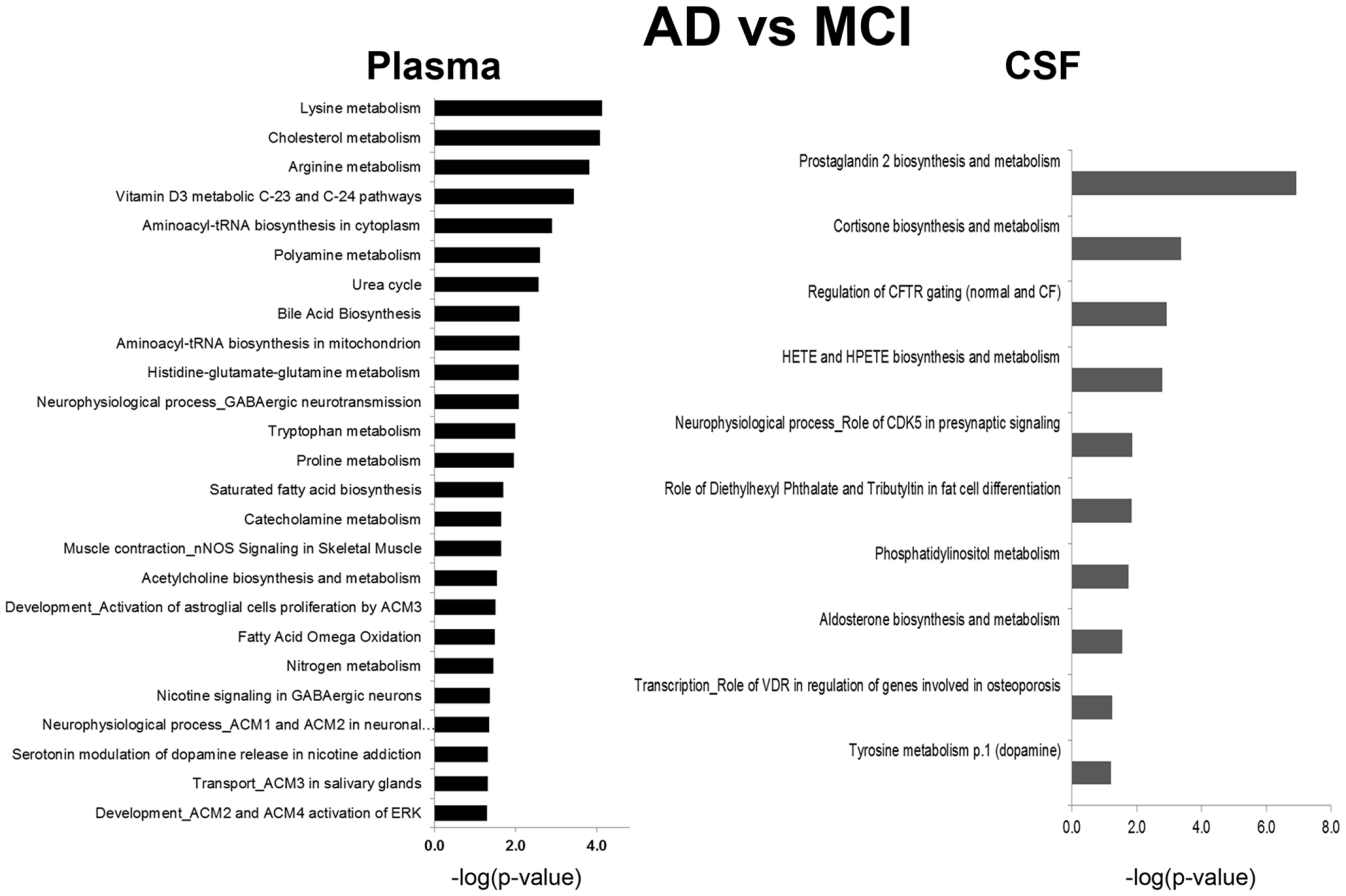
Altered metabolic pathways and process networks specifically affected either in plasma or in CSF of AD vs. MCI subjects. The significance of the pathways was evaluated using *P* values and false discovery rate <0.05. GABA - γ-Aminobutyric acid; nNOS – neuronal nitric oxide synthase; CFTR - cystic fibrosis transmembrane conductance regulator; VDR - vitamin D receptor; HETE - hydroxyeicosatetraenoic acid; HPETE - hydroperoxyeicosatetraenoic acid.

Comparison of metabolites in biofluids from AD vs. MCI subjects identified differences in 44 (*P*≤0.05) plasma and 31 (*P*≤0.05) CSF metabolites (Tables S8 and S9, respectively). Among metabolites affected to the same extent in plasma and CSF were 2-methylbutyrylglycine and amino acids tyrosine, alanine and leucine (Tables S8 and S9). Based on the changes in metabolites, we identified 25 (*P*≤0.05) pathways in plasma and 10 (*P*≤0.05) in CSF that significantly differed between MCI and AD (Fig. 6). The ten altered pathways in CSF were related to neurotransmitter, lipids, and sterol metabolism, with prostaglandin 2 (PGE2) and cortisone biosynthesis and metabolism were the most affected (Fig. 6). The major metabolites altered in these pathways were prostaglandins (PG G2, PG J2), hydrocortisone, and tetrahydrocortisone. Significantly larger amount of pathways was affected in plasma compared to CSF of AD vs. MCI patients (Fig. 6). These pathways were related to amino acid, cholesterol, lipids, neurotransmitter, and mitochondrial biosynthesis and metabolism.

**Figure 7:**
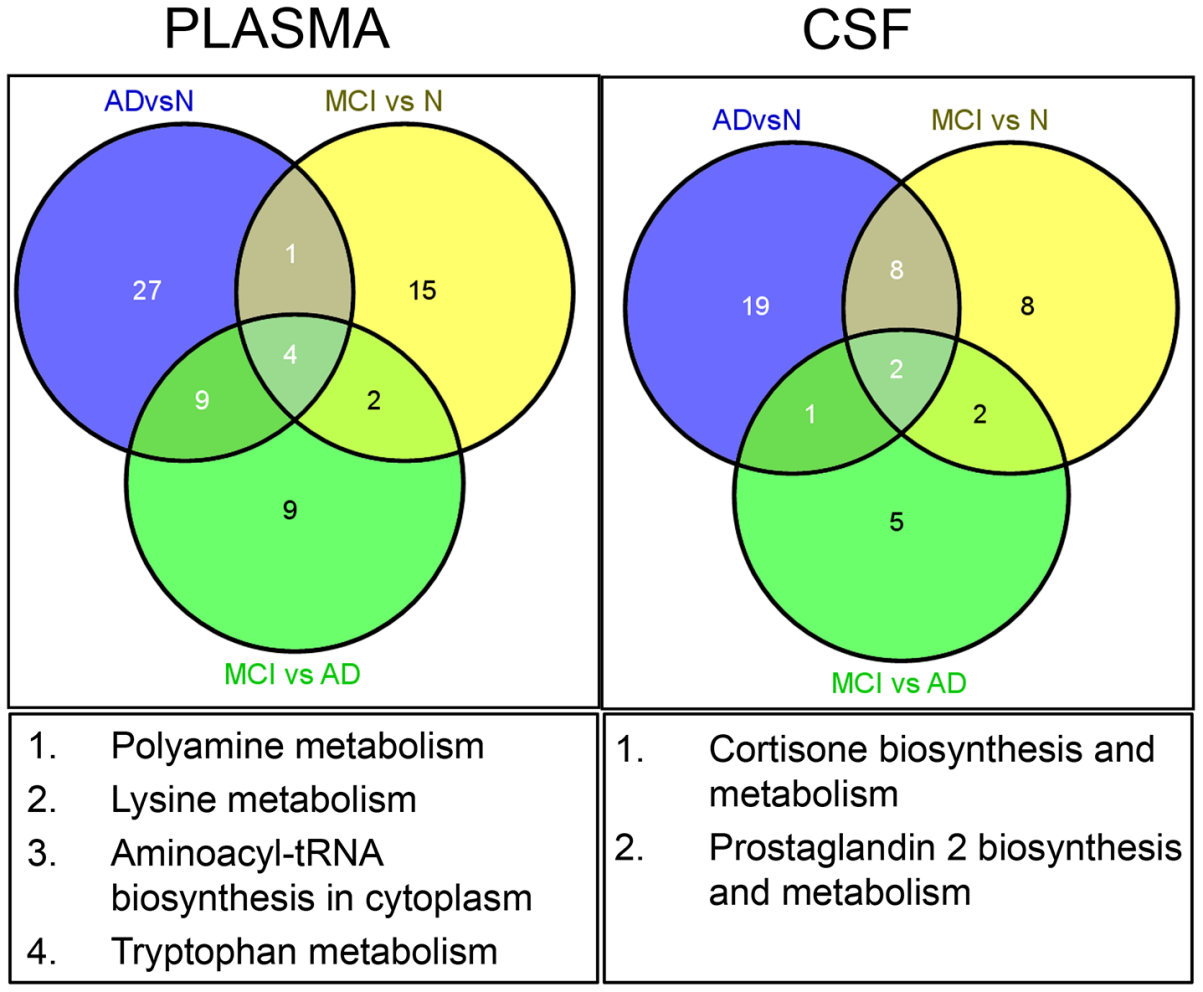
Venn diagram illustrating shared and uniquely affected pathways in plasma and CSF of MCI, AD and CN individuals. Common pathways are defined.

A Venn diagram depicts the specific pathways in plasma and CSF, which cross-sectionally differentiate the three diagnostic groups with respect to the disease severity (Fig. 7). Four common pathways affected in plasma include polyamine metabolism, lysine metabolism, aminoacyl-tRNA biosynthesis in the cytoplasm, and tryptophan metabolism. Two pathways affected in CSF are related to cortisone and PGE2 biosynthesis and metabolism (Fig. 7).

## Discussion

Metabolomics is a rapidly emerging “omics” that establishes disease-specific signatures of perturbations in hundreds of metabolites, reflecting alterations in multiple networks affected in the disease. Application of metabolomics for the diagnosis of AD is very attractive. Profiling in plasma and CSF could be used to establish metabolic signatures for the purposes of an accurate diagnosis, including the early clinical (e.g., MCI) or preclinical stages, or within individuals to monitor disease progression and therapeutic efficacy. Metabolomics can be conducted in biofluids, such as plasma or CSF, with high throughput and relatively low cost. Recently, metabolomic profiling was used to assess cross-sectional alterations in either CSF or plasma samples from individuals with different clinical severity of AD [17], [18], [26], [28]–[31]. However, these studies used diverse metabolomics platforms, biofluids (CSF or plasma), and differed in the range of identified metabolites, thus limiting efforts of comparing the results. In the present study, we applied a non-targeted mass spectrometry–based metabolomic profiling to determine global changes in metabolites and various putative metabolic pathways in CSF and plasma from the same individuals in relationship to AD progression. To our knowledge, this is the first study that (1) evaluates progressive metabolic changes in CN, MCI and AD subjects in both CSF and plasma, (2) establishes to what extent metabolic changes in lumbar CSF are reflected in plasma, (3) identifies unique and common metabolic pathways specifically affected by AD severity in plasma and CSF, and (4) validates plasma as a reliable biofluid for metabolic studies of brain-related disorders. We found that approximately 30% of the metabolic pathways altered in the CSF in MCI patients vs. CN, and ∼60% in AD patients vs. CN, were also affected in plasma from the same individuals (Fig. 4, 5). The number of affected pathways in CSF and plasma increased with disease severity. Thus, in AD patients the total of affected pathways increased by 50% in CSF and doubled in plasma compared to MCI patients (Fig. 4, 5). However, in MCI and AD individuals the number of affected pathways was always greater in plasma, thus reflecting changes in organs other than brain that are associated with AD (Fig. 4–7). Our data demonstrate that signatures in CSF and plasma have significant overlap, and most of the pathways affected early in MCI continue to be altered in AD subjects. In both CSF and plasma in MCI and AD groups the perturbed canonical pathways included those related to energy metabolism and mitochondrial function; lipid biosynthesis, trafficking and metabolism; amino acid biosynthesis and metabolism; neurotransmitter biosynthesis and metabolism; and hormone biosynthesis and metabolism (Fig. 4–7). However, we also identified pathways that were specifically affected in either plasma or CSF (Fig. 4–7).

One of the prominent dysfunctions in AD is a progressive failure of neuronal networks and neurotransmitter systems. Results of the extensive studies in multiple animal and cellular models of AD suggest that synaptic malfunction and synaptic loss occur prior to the development of Aβ plaques and neurofibrillary tangles [41]. These synaptic alterations are directly associated with deteriorated synaptic strength and synaptic plasticity, including long-term potentiation [41]. Acetylcholine, noradrenalin, dopamine and serotonin neurotransmitter systems are primarily affected in AD with subsequent loss of associated neurons [42]. Consistent with that, we have found prominent, early changes in tryptophan biosynthesis in both CSF and plasma of MCI and AD patients (Fig. 4–6). Tryptophan is a precursor for serotonin, melatonin, and niacin synthesis [43]–[45]. Therefore, not surprisingly, we found alterations in the serotonin/melatonin pathway in CSF of both MCI and AD patients, and in plasma of MCI individuals (Fig. 4, 5). These data are in agreement with recent studies indicating that loss of serotonergic neurons correlates with AD severity, memory impairment, and neuropsychiatric symptoms [42], [46], and that melatonin protects against Aβ toxicity in cellular and animals models of AD [47], [48]. Additional significant changes in neurotransmitter metabolism were observed in the acetylcholine pathway in CSF from AD individuals and in gamma amino butyric acid (GABA) pathway in plasma from MCI patients (Fig. 4, 5). This is in agreement with the previously reported changes detected in post-mortem CSF samples from patients with confirmed AD using targeted metabolomics [26]. We also found that in addition to tryptophan, multiple amino acid metabolic pathways were progressively affected in CSF and plasma in AD and MCI patients compared to CN (Fig. 4, 5). While only L-arginine and tryptophan pathways were altered in both plasma and CSF of MCI patients (Fig. 4), the number of pathways equally affected in CSF and plasma of AD patients considerably increased and included beta-alanine, aspartate and aspargine, alanine, L-cysteine, L-methionine, methionine-cysteine-glutamate along with L-arginine and lysine metabolism (Fig. 5). Our findings are in a good agreement with alterations in amino acids measured in CSF of AD patients using variety of methods [26], [28], [30], [31], [49]. It is important to note that pattern of alterations in amino acid pathways in plasma was very similar to the observed in CSF in both MCI and AD cohorts (Fig. 4, 5). However, significant alterations in the lysine pathway were detected only in the plasma in MCI and AD individuals (Fig. 4, 5). It is known that lysine being a strictly ketogenic amino acid, is also required for the synthesis of L-carnitine. L-Carnitine is the only transporter of fatty acids to mitochondria to be metabolized with production of energy. Indeed, previous study demonstrated that levels of carnitine were lower in CSF from MIC-AD and AD patients than in CSF from non-AD subjects [28]. In our study, perturbations of the lysine metabolic pathway most accurately differentiated CN from the MCI and AD groups in plasma (Fig. 7).

Previous metabolomics studies detected changes in the levels of the neurotransmitter norepinephrine (NE) and its major metabolite 3-methoxy-4-hydroxy phenylglycol (MHPG) involved in noradrenalin neurotransmitter system [50]. Loss of NE would be expected as a result of neuronal loss in locus coeruleus established in AD patients [51]. Interestingly, Czech et al reported increased levels of NE in CSF of AD subjects suggesting a compensatory mechanism where surviving neurons have higher secretion of NE [30]. However, another study reported the opposite – a decrease in NE levels and an increase in MHPG in the CSF of AD subjects [26]. We were able to identify both metabolites in the CSF of CN, MCI and AD subjects; however, levels of these metabolites were not significantly changed between our study groups. It will be interesting to determine whether the discrepancy between the results could partially be explained by the impact of the post mortem changes or the origin of the CSF samples – ventricular post-mortem CSF [26] vs. lumbar, as in our study and [30], or the different metabolomics platforms utilized in all of the above-mentioned studies.

Two additional pathways that were significantly and specifically affected in plasma by AD severity included polyamine metabolism and aminoacyl-tRNA biosynthesis in the cytoplasm (Fig. 7). Alterations in the levels of polyamines found in the brain tissue of AD patients have been linked to the abnormal regulation of calcium flux, glutamate receptor function, and excitotoxicity [52]. The major metabolites affected in that pathway in plasma of MCI and AD individuals included, but were not limited to, arginine, tryptophan, proline, lysine, glutamine, GABA, and urea. Aminoacyl-tRNA synthetases (AARS) and translation factors are key enzymes required for protein biosynthesis. The exact mechanisms associated with altered AARS pathway in AD remain unknown. However, recently AARS was linked to the biosynthesis of the dinucleotide polyphosphates [53], which play an important role as neurotransmitters, stimulate GABA release in peripheral and central nervous system, and are involved in the response to oxidative stress and metabolic changes [54]–[56].

A number of studies have proposed the role for mitochondrial dysfunction in the early pathogenesis of AD [25], [57]–[61]. We have previously demonstrated the presence of metabolic signatures of energetic stress and mitochondrial dysfunction in brain tissue from three transgenic mouse models of familial AD (FAD) [25]. Consistent with our previous findings, the current study also identified multiple pathways related to energy metabolism and mitochondrial function that were already significantly affected in MCI cohort. The TCA (Krebs) cycle was most affected in both the CSF and plasma of MCI and AD patients (Fig. 4, 5). We also found significant alterations in saturated fatty acid metabolism in the plasma of MCI patients and in the CSF of AD patients, and fatty acid omega oxidation in the plasma of AD patients (Fig. 4–6). Additional pathways related to altered brain energetics included pyruvate, mitochondrial ketone bodies, glycolysis and gluconeogenesis, and were affected in CSF and/or plasma from MCI and AD patients supporting the role for mitochondrial dysfunction in early AD. Among additional pathways correlated with AD progression was the urea cycle [49]. We have found that alterations in urea cycle were detected only in the CSF of MCI subjects but in both, CSF and plasma of AD patients (Fig. 4–7). Changes in the urea cycle correlate well with previously reported changes in body fluids of AD patients [49] and could be used to discriminate between those MCI patients who do and do not progress to AD (Fig. 7).

An additional strength of our study is the application of UPLC-ToF-MS – based metabolomics, which allows for the detection of relative changes in multiple metabolites associated with AD clinical severity, including lipids [36], [62]. Utilization of MS in conjunction with gas chromatography (GC) and high performance liquid chromatography (HPLC) systems has recently become very popular. However, application of UPLC-MS has an advantage in that it does not require deconjugation and derivatization steps before analysis [63], [64]. Moreover, samples are processed at low temperatures relatively to the temperatures used with GC-MS, which allows for the detection of labile sterols that at high temperatures could be unstable [65]. Previous studies identified perturbations in the levels of phosphatidylcholine, plasmalogens, sphingomyelins and sterols in plasma of subjects with AD [16]–[19], [31], [66]–[68]. A decrease in desmosterol and the desmosterol/cholesterol plasma ratio measured using metabolomics technology was also proposed as a sensitive marker for AD [29]. However, it is well known that AD patients lose weight, which results in lower lipid levels. Therefore, it is important to determine the specificity of lipid subclasses affected with AD severity rather than an overall lipid flux. In agreement with the previous studies, we have found that pathways related to cholesterol biosynthesis and metabolism, cholesterol and sphingolipids transport, lipid metabolism and other phospholipid and plasmalogen pathways were significantly altered in both CSF and plasma from MCI and AD patients (Fig. 4, 5). The number of altered pathways related to lipid biosynthesis and metabolism was progressively increased in AD patients relative to MCI (Fig. 5). Discrimination between MCI and AD patients demonstrates that metabolic signature of altered cholesterol metabolism was prevalent in plasma samples and of altered phosphatidylinositol metabolism in CSF (Fig. 6). Moreover, our study identified CSF PGE2 biosynthesis and metabolism as one of the key pathways that varied with AD severity (Fig. 7). Implication of PGE2 in neural injury in AD is well documented, and includes modulation of protein-lipid interactions, trans-membrane and trans-synaptic signaling [69]. It was shown that levels of PGE2 measured in the CSF of control, MCI and AD patients enrolled in the longitudinal study inversely correlate with AD severity: PGE2 was higher in patients with mild memory impairment, but lower in those with more advanced AD [70].

Disorder in the hypothalamic-pituitary-adrenal (HPA) axis with increased cortisol levels in CSF and plasma is also well established for AD patients; and increased cortisol levels in CSF from AD patients have been recently demonstrated using metabolomic profiling [30]. Our data confirmed that the pathway related to the cortisol biosynthesis from cholesterol was significantly affected in both CSF and plasma from AD patients (Fig. 5). However, we also found that cortisone biosynthesis and metabolism was among the pathways that, along with PGE2, most accurately separated the clinical groups in CSF (Fig. 6). Among pathways that were uniquely affected in plasma of AD patients were those related to obesity and type II diabetes mellitus (Fig. 5). This is an important observation taking in consideration the data demonstrating that type II diabetes mellitus is associated with an increased risk of cognitive dysfunction and dementia, and needs to be explored in future studies [71].

Together, the present results, utilizing comprehensive metabolic profiling, in AD and MCI subjects confirmed previously reported observations and identified novel metabolic signatures in CSF and plasma that vary with the clinical severity of AD. Interestingly, the fact that patients were on multiple medications did not impact our ability to obtain data with significant differences between groups (Table S10). This could partially be explained by the overlap of the medication among all three groups (Table S10, bold font depicts same medication) or the fact that medication does not significantly affect the disease progression. Further, current metabolomics approaches, in addition to measuring metabolites originated from endogenous cellular metabolism, also detect those derived exogenously from drugs, food, and cosmetics. However, we were still able to observe robust group separation supporting high sensitivity of the approach. A strength of the study is in utilizing UPLC-ToF-MS to detect changes in broad variety of metabolites that reflect the complexity of metabolic networks altered in AD. The accuracy of our findings was also enhanced by the precise and consistent selection of participants in order to closely match experimental groups on demographic factors. However, limitations of the study also warrant consideration. One limitation was the small sample size of 15 participants per clinical group. While we still achieved robust group separation, additional studies are necessary to validate our findings in larger cohorts. It will also be important to examine the effect of sex, as the participants included in this analysis were primarily men. Lastly, future studies will need to assess the specific changes in identified pathways to shed light on disease mechanisms along with assaying the longitudinal changes in the pathways and metabolites as indicators of disease progression, especially at the early pre-clinical stages.

Taken together, our studies demonstrate that overlapping alterations in several known and unknown metabolites and various putative metabolic pathways could be detected using non-targeted mass spectrometry–based comprehensive metabolic profiling in CSF and plasma in MCI and AD individuals in respect to AD severity. The agreement of our data with previously reported changes in metabolites and metabolic pathways associated with AD or MCI and identified using multiple analytical approaches offers further support for metabolomics analysis of plasma and CSF samples for AD diagnosis. Our results suggest that additional studies with targeted metabolomics could identify specific panels of metabolites. Furthermore, the significant similarity of affected pathways based on changes in plasma and CSF metabolites and canonical pathways supports the notion that plasma closely depicts biochemical fingerprints of brain changes in AD and MCI individuals. Our data validate plasma as reliable source for metabolomic profiling and suggest that metabolomics is a valuable tool for the identification of molecular mechanisms involved in the etiology of AD and novel therapeutic targets.

## Supporting Information

Table S1 **Altered metabolites in plasma of AD, MCI and CN patients.** Data was generated using 1 way ANOVA (p<0.05). FC – fold change between indicated groups; MCI – mild cognitive impairment; AD – Alzheimer’s Disease; CN – cognitively normal. Metabolites detected and identified in all three study groups are highlighted in bold font. Compound identification codes: CAS- Chemical Abstracts Service; KEGG- Kyoto Encyclopedia of Genes and Genomes; HMP- Human Metabolome Project; LMP ID- nonpolar lipid metabolite IDs.
(XLSX)

Table S2 **Altered metabolites in CSF in AD, MCI and CN patients.** Data was generated using 1 way ANOVA (p<0.05). FC – fold change between indicated groups; MCI – mild cognitive impairment; AD – Alzheimer’s Disease; CN – cognitively normal. Metabolites detected and identified in all three study groups are highlighted in bold font. Compound identification codes: CAS- Chemical Abstracts Service; KEGG- Kyoto Encyclopedia of Genes and Genomes; HMP- Human Metabolome Project; LMP ID- nonpolar lipid metabolite IDs.
(XLSX)

Table S3 **Identification and validation of plasma and CSF metabolite standards using UPLC-ToF MS.** Compound identification codes: CAS- Chemical Abstracts Service; KEGG- Kyoto Encyclopedia of Genes and Genomes.
(XLSX)

Table S4 **Metabolites altered in plasma of MCI vs. CN.** Data was generated using unpaired t-test. FC – fold change between indicated groups; MCI – mild cognitive impairment; CN – cognitively normal. Compound identification codes: CAS- Chemical Abstracts Service; KEGG- Kyoto Encyclopedia of Genes and Genomes; HMP- Human Metabolome Project; LMP ID- nonpolar lipid metabolite IDs.
(XLSX)

Table S5 **Metabolites altered in CSF of MCI vs. CN.** Data was generated using unpaired t-test. FC – fold change between indicated groups; MCI – mild cognitive impairment; CN – cognitively normal. Compound identification codes: CAS- Chemical Abstracts Service; KEGG- Kyoto Encyclopedia of Genes and Genomes; HMP- Human Metabolome Project; LMP ID- nonpolar lipid metabolite IDs.
(XLSX)

Table S6 **Metabolites altered in plasma of AD vs. CN. Data was generated using unpaired t-test.** FC – fold change between indicated groups; AD – Alzheimer’s Dsiease; CN – cognitively normal. Compound identification codes: CAS- Chemical Abstracts Service; KEGG- Kyoto Encyclopedia of Genes and Genomes; HMP- Human Metabolome Project; LMP ID- nonpolar lipid metabolite IDs.
(XLSX)

Table S7 **Metabolites altered in CSF of AD vs. CN.** Data was generated using unpaired t-test. FC – fold change between indicated groups; AD – Alzheimer’s Dsiease; CN – cognitively normal. Compound identification codes: CAS- Chemical Abstracts Service; KEGG- Kyoto Encyclopedia of Genes and Genomes; HMP- Human Metabolome Project; LMP ID- nonpolar lipid metabolite IDs.
(XLSX)

Table S8 **Altered metabolites in plasma of AD vs. MCI.** Data was generated using unpaired t-test. FC – fold change between indicated groups; AD – Alzheimer’s Dsiease; MCI – mild cognitive impairment. Compound identification codes: CAS- Chemical Abstracts Service; KEGG- Kyoto Encyclopedia of Genes and Genomes; HMP- Human Metabolome Project; LMP ID- nonpolar lipid metabolite IDs.
(XLSX)

Table S9 **Altered metabolites in CSF of AD vs. MCI.** Data was generated using unpaired t-test. FC – fold change between indicated groups; AD – Alzheimer’s Dsiease; MCI – mild cognitive impairment. Compound identification codes: CAS- Chemical Abstracts Service; KEGG- Kyoto Encyclopedia of Genes and Genomes; HMP- Human Metabolome Project; LMP ID- nonpolar lipid metabolite IDs.
(XLSX)

Table S10 **List of medication for participants in CN, MCI and AD groups.** Table represents medications used by each study participant at the time of sample collection. Similar medication is highlighted in bold. MCI – mild cognitive impairment; CN – cognitively normal, AD – Alzheimer’s Disease.
(XLSX)
